# Qualifications and Administrators

**Published:** 1918-11-16

**Authors:** 


### THE MEDICAL SERVICE OF THE R.A.F.

### Qualifications and Administrators.

The joint letter of the Members' Medical Advisory Committee and the Chairman of the Parliamentary Air Committee in *The Times* of Nov. 8 is a curious contribution to medical literature. In essence it is a protest against the appointment of Col. Fell to administer the Medical Service of the R.A.F., not because the writers consider Colonel Fell a poor administrator—they acknowledge he has had great experience in administrative capacities—but because he is not known to be in the first rank as a physician. The business of an administrator is to administer. That is what he is asked to do, and to do well. A man may be one of the best surgeons in the world, or the highest authority on the physiology of blood pressures, and yet be useless as an administrator. In fact, it is quite possible that the very concentration of his powers on special and limited subjects that has made a man an authority on those subjects may have prevented him acquiring or developing the gifts and the power to judge of men that make a man a good administrator. "Put shortly, the whole object of this Committee in formulating their scheme of an Air Medical Service was that promotion should be by medical efficiency, and not, as in the R.A.M.C., by administrative capacity."

In other words, We meant men to be made administrators not because they could administer, but because they were good physicians or surgeons.

And thus, "in our opinion the ground was laid for a Medical Service superior to that of any existing Force."

There was a little doubt in the minds of the Committee as to the capacity of the Medical Administrator of the Air Force that this system of selection would obtain, so "a Medical Administrative Committee was instituted in order to assist the Medical Administrator," in case, in spite of his scientific attainments, he should prove incompetent as an administrator. It comes to taking a first-class scientific man from work he can do well, putting him to do work he can only do poorly, and bolstering him up with a committee. In the next paragraph it is complacently suggested that the new Civilian Medical Service, when it comes to be, will be much better administered on such lines as these than on the lines of the R.A.M.C. Throughout the letter there is an extraordinary confusion of scientific administration with administration by scientists.

If the directorate of a furniture-making firm promoted the best artist in the designing-rooms to the post of general manager because he was a good artist, if a chemical manufacturer promoted his most scientific analytical chemist to a post of administrative importance because he was an authority on experimental chemistry, none would be surprised at the bankruptcy of either business.

The reward of the physician, of the artist, or of the chemist should be as great or greater than the reward of the administrator, but to make a man an administrator to reward his success in other work without consideration of his capacity as an administrator sounds as fatuous as to refuse the services of a capable administrator as administrator because he is not a first-class physician, artist, or chemist. The failure of the new Civilian Medical Service is assured in advance if such are to be the lines of its administration. Few will wonder that practical men of affairs fail to see eye to eye with the Members' Medical Advisory Committee. A medical administrator must have a good general knowledge of medicine and surgery and the conditions of medical treatment, but it is not necessary that he should have the high skill of a great surgeon, the acumen in diagnosis of a great physician, because in his work he will not have opportunity to use either. If it should happen that a man be great as a surgeon and great as an administrator, it may be left to the circumstances of the time to decide from which set of duties his dual ability can best be spared. He cannot do both sorts of work on the great scale.

